# Nosocomial Candidemia; Risk Factors and Prognosis Revisited; 11 Years Experience from a Norwegian Secondary Hospital

**DOI:** 10.1371/journal.pone.0103916

**Published:** 2014-07-31

**Authors:** Jan-Erik Berdal, Rolf Haagensen, Trond Ranheim, Jørgen V. Bjørnholt

**Affiliations:** 1 Department of Infectious Diseases, Akershus University Hospital, Nordbyhagen, Norway; 2 Department of Anaesthesiology, Akershus University Hospital, Nordbyhagen, Norway; 3 Department of Microbiology, Akershus University Hospital, Nordbyhagen, Norway; 4 Department of Infectious Disease Epidemiology Norwegian Institute of Public Health, Oslo, Norway; California Department of Public Health, United States of America

## Abstract

The aim of the study was to review the epidemiology and prognosis of candidemia in a secondary hospital, and to examine the intra-hospital distribution of candidemia patients. Study design is a retrospective cohort study. Trough 2002–2012, 110 cases of candidemia were diagnosed, giving an incidence of 2, 6/100000 citizens/year. Overall prognosis of candidemia was dismal, with a 30 days case fatality rate of 49% and one year case fatality rate of 64%. Candidemia was a terminal event in 55% of 30 days non-survivors, defined as Candida blood cultures reported positive on the day of death or thereafter (39%), or treatment refrained due to hopeless short-term prognosis (16%). In terminal event candidemias, advanced or incurable cancer was present in 29%. Non-survivors at 30 days were 9 years (median) older than survivors. In 30 days survivors, candidemia was not recognised before discharge in 13% of cases. No treatment were given and no deaths or complications were observed in this group. Candidemia patients were grouped into 8 patient categories: Abdominal surgery (35%), urology (13%), other surgery (11%), pneumonia (13%), haematological malignancy (7%), intravenous drug abuse (4%), other medical (15%), and new-borns (3%). Candidemia was diagnosed while admitted in the ICU in 46% of patients. Urology related cases were all diagnosed in the general ward. Multiple surgical procedures were done in 60% of abdominal surgery patients. Antibiotics were administered prior to candidemia in 87% of patients, with median duration 17 (1–108) days. Neutropenia was less common than expected in patients with candidemia (8/105) and closely associated to haematological malignancy (6/8). Compared with previous national figures the epidemiology of invasive candidiasis seems not to have changed over the last decade.

## Introduction

The epidemiology of invasive candidasis varies between regions and countries, with higher rates in north-America than in Europe, and an increasing north-south gradient observed in both [1]. Within Scandinavia an unexplained difference exists with high incidence in Denmark and low incidence in the other Nordic countries [2]–[5]. Mortality associated with candidemias has been reported in the 40–70% range across a number of studies [6]–[8]. However, unbiased attributable candidemia mortality is difficult to establish, and the effect of treatment in reducing overall mortality, especially in low prevalence settings remains unclear. Risk factors such as central line catheters, parenteral nutrition, steroids, antibiotic usage, renal replacement therapy and diabetes are well recognised but present too often to discriminate patients at risk, and proposed prediction rules are of limited value in low-incidence settings [9]–[12]. Patients undergoing multiple abdominal surgery, suffering from haematological malignancies, or residing in ICU's are recognized risk groups [10], [13], but otherwise data on the intra-hospital epidemiology of candidemia is scarce. The aims of study were threefold 1) To investigate the epidemiology of candidemia from 2002–2012 compared with Norwegian national figures for the previous 13 year period [14] 2) To investigate overall outcome and elucidate attributable mortality looking into differences between 30 days survivors and non-survivors, as well as quantifying the risk factors antibiotic usage and surgeries prior to candidemia and 3) To investigate differences in the in-hospital epidemiology of candidemia across wards and specialities, hypothesising candidemia patients would be found predominantly in a limited number of clinical recognisable groups. Exploring this, we attempted grouping candidemia patients into defined and recognisable categories, and investigated differences in candidemia incidence in medical and selected surgical departments. The study was performed in a large secondary referral hospital with a catchment area covering 7–10% of the Norwegian population, encompassing a broad range of medical and surgical specialities.

## Materials and Methods

### Design

Retrospective cohort study including all patients ≥18 years of age admitted from 01.01.2002 until 31.12.2012. Four patient's ≤18 years of age and 1 patient with missing data (5/110) were included in the calculation of population-based incidence, but not in the other analyses.

### Setting

Akershus University Hospital is a 650 bed secondary referral hospital, with a catchment area comprising 320000 inhabitants up to 2010 thereafter 460 000 inhabitants or almost 10% of the Norwegian population. All specialities except neurosurgery, heart surgery and transplant surgery are covered. The haematology department is among the largest in Norway, but leukaemia induction and bone marrow transplant therapies are not performed. From 2005–2012, 2193 patients (0, 68% of total) were discharged from the hospital with primary or co-diagnosis neutropenia (ICD-D70). The ICU is a combined medical and surgical ICU with 10 beds, where the majority of patients are in need of ventilator support (2200 ventilator days 2012).

### Microbiological sampling

During the study period 251799 blood culture bottles were incubated, with median annual positive rate of 6, 8% (range 5, 4–7, 2) over the study period. Candida albicans was in 2011 and 2012 ranked the 12 and 15 most common findings in blood cultures with 18 and 10 unique patients as compared with 235 and 265 for the number one *E.coli*. Hospital blood sampling routines requires two 10 ml bottles of blood drawn from 2 separate sites (total 40 ml). The BACTEC9240 system was used until 2008, thereafter the BACTEC FX20. All positive cultures were sent to the national reference laboratory for confirmation and further identification and susceptibility testing. An episode was considered unique if single, or if not, occurring ≥21 days apart or caused by different species. Catheter related candidemia was defined according to IDSA guidelines [15].

### Patient identification and data extraction

Candidemia cases were identified in the database of the department of microbiology. Relevant study data were extracted from the electronic patient charts. The following data were recorded: age, gender, Candida species, admission ward, ICU stay during shortly before or after (<5 days) a positive Candida blood culture, diagnosis, Simplified Acute Physiology Score II (SAPS II) score (ICU patients), number and type of surgical procedures, antibiotic days prior to positive Candida blood culture, central venous lines, positive central venous line catheter cultures, parenteral nutrition, neutropenia, diabetes, immunosuppression defined as metastatic cancer, immunosuppressive medication other than steroids (recorded separately), and in hospital and end of study mortality. Two investigators categorised patients into 6 major diagnostic categories, 3 surgical and 3 medical according to organ responsible for hospitalisation or speciality involved in management, or with other clear defining characteristics such as preterm or intravenous drug abuse. In ambiguous cases criteria were re-examined and a consensus reached. In both surgical and medical patients a category “other” had to be created for groups with multiple diagnoses and diverse major surgery, constituting 28/105 (27%) of the cohort.

### Statistical analysis

Incidence of unique episodes was calculated correcting changes in population, especially the increase in catchment area at the end of study period. Incidence confidence intervals were calculated according to the Byar approximation [16]. Standard descriptive statistics were performed using mean (SD) or median (range) as appropriate. The associations between time to death from last positive blood culture and diagnostic categories were investigated by Kaplan-Meyer survival plots and log rank tests. P-values are two-tailed. Statistical analyses were performed using StatView© 5.0 SAS Institute Inc.

### Ethical considerations

The study protocol was presented to the Regional Ethical Committee of South-Eastern Norway (REK) and approved as not requiring patient informed consent. The Internal Privacy Ombudsman of Akershus University Hospital approved the study, and patient information was anonymized and de-identified prior to analysis.

## Results

**Figure 1:**
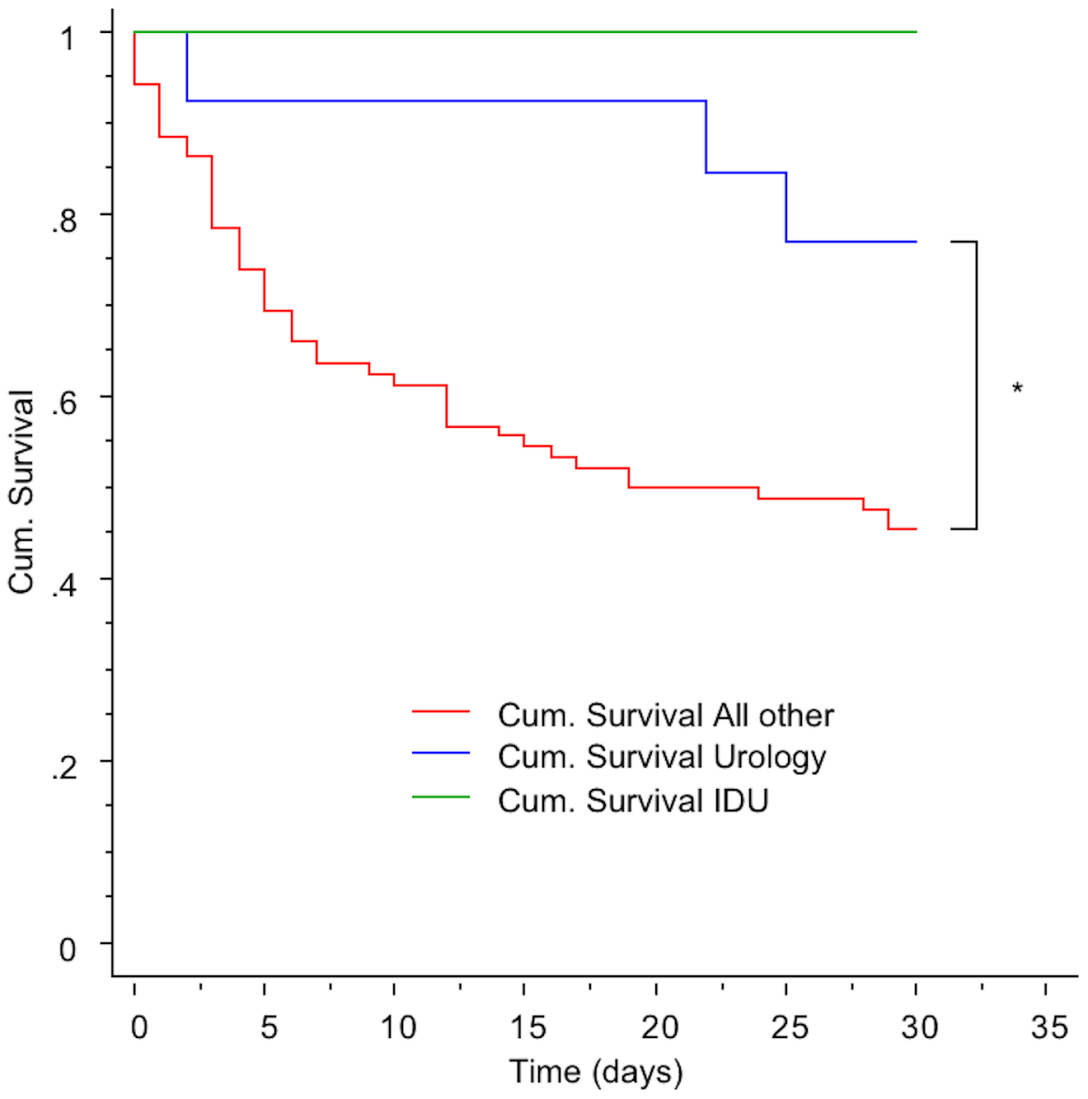
Thirty days cummulative survival according to clinical recognisable categories. IDU and Urology compated to all other categories (Other surgery, Abdominal surgery, Other medical, Pneumonia, Hematology). * Log rank test p = 0,0425.

110 individual patients with candidemia were identified over the 11 years period, several with multiple positive cultures, giving an incidence of 2, 6/100000 citizens/year of unique episodes. Candida albicans was by far most prevalent, species distribution and susceptibility patterns are shown in table 1. Candidemia incidence proportions per 1000 discharged patients from general Medical, Haematological, Gastro-surgical, and Urology- wards are shown in table 2. Patients received anti-fungal therapy according to susceptibility results, except in 28 cases were blood cultures became positive on the day of death or thereafter (n = 20), or the candidemia was considered a terminal event and treatment refrained (n = 8). Another 7 patients were discharged untreated before candidemia was recognized, all cases without clinical consequences (Table 3). Fluconazol was the most used empirical antifungal, with an increased use of ecchinocandins empirically towards the end of the period, rapidly de-escalating to fluconazole in stable patients. Except in a few selected haematological patients, antifungals were not used for prophylaxis. Thirty days case fatality rate was 49%, and one-year mortality was 64%. Analysing survival according to clinical categories revealed three distinct patterns, with the clinical categories Intravenous Drug Use (IDU) (30 days case fatality 0) and Urology (30 days case fatality 0,29) having significant lesser case fatality than all other categories (30 days case fatality 0,50–0,62). A Kaplan Meier plot is depicted in figure 1. Follow-up for 90 and 1 year did not change this pattern (data not shown). Advanced or incurable cancer was present in 15 patients in the 30 days non survivors group, and in 5 surviving 30 days, median survival in this latter group was 82 days, and only one survived more than a year (range 38–485 days). Median age in non-survivors at 30 days was 74 years (range 31–93) versus 65 years (range 24–92) in survivors. Distribution of antibiotic days prior to candidemia and other established risk factors are shown in table 4. Mean SAPS II score in the ICU candedima patients was 49 (SD 15). Catheter related candidemia was diagnosed in13 patients (12%), evenly distributed between the clinical recognisable category Pneumonia and Haematological. Within the Gastro-surgery category perforated viscus or anastomosis leakage were present in18/37 (49%) and pancreatitis in 5/37 (13%) patients. 22/37 (59%) of patients underwent at least 2 abdominal surgical procedures before or shortly after occurrence of candidemia. In 10/37 (27%) patients between 3 and 8 surgical procedures were performed. Among the patients categorised in the Urology category 11/14 (79%) had obstructive uropathy with a foreign body, 9/14 (64%) either replaced or got a new nephrostomy in close temporal relation to the candidemia.

**Table 1:** Species distribution and in vitro antifungal susceptibility in 112 Candida blood culture isolates 2002–2012.

Candida species	Number	Fluconazole S/I/R	Vorikonazole S/I/R	AmphotericinB S/I/R	Anidulafungin S/I/R	Micafungin S/I/R
*C. albicans*	85	85/0/0	81/0/0	85/0/0	40/0/1	27/0/3
*C. tropicalis*	10	0/10/0	7/0/1	9/0/1	2/0/0	-
*C. parapsilosis*	10	7/2/1	9/0/1	9/0/1	0/5/0	0/5/0
*C. glabrata*	7	0/6/1	-	7/0/0	4/0/0	4/0/0

S/I/R categorisation according to EUCAST clinical breakpoints (v 6.1, available 2013-03-11) www.eucast.org.Some patients yielded more than one isolate, see text. All isolates are not tested for all antifungals.

**Table 2:** Candidemia incidence proportion (95% CI) per 1000 admitted per year, for patients above 18 years, in selected departments* 2002–2012.

	Department of gastrosurgery	Department of urology	Medical incl. Hematological department	All departments
Incidence proportion (95% CI)	0,67 (0,47–0,94)	0,46 (0,24–0,88)	0,27 (0,20–0,34)	0,23 (0,19–0,28)

*data for department of admittance are not available for all department throughout the entire observational period.

95% CI calculated according to [15].

**Table 3:** Clinical characteristics according to 30 days outcome of candidemia.

	Survivors	Non-survivors
Number	54	51
Age (years)*	65 (24–92)	74 (31–93)
Terminal event:	NA	28 (55)
- Culture positive on day of death or later	NA	20 (39)
- Treatment refrained	NA	8 (16)
- Advanced incurable cancer	NA	15 (29)
- ICUadmittance	NA	14 (27)
Advanced incurable cancer (all)	5 (9)	16 (31)
Not recognised before discharge	7 (13)	NA
ICUadmittance	22 (41)	24 (47)

Values represent n (%) except * given as median (range).

**Table 4:** Risk factors/descriptives according to clinical categories.

	Gastrosurgery	Urological	Pneumonia	Haematological	IDU	Othermedical	Othersurgery	All
*N (%)*	*37 (35)*	*14 (13)*	*14 (13)*	*8 (7,6)*	*4 (3,8)*	*16 (15)*	*12 (11)*	*105 (100)*
Age (years)#	74 (24–84)	77 (71–82)	73 (47–88)	64 (58–76)	43 (31–63)	68 (25–82)	61 (31–87)	70 (24–78)
Male sex (%)	59,5	85,7	78,6	50,0	100	62,5	41,7	64,8
Antibiotics*	33 (89)	12 (86)	14 (100)	8 (100)	1 (25)	11 (69)	10 (83)	91 (87)
Antibiotics(days)#	22 (0–108)	16 (0–70)	17 (3–32)	13 (1–67)	2 (0–2)	11 (0–88)	15 (0–42)	15 (0–108)
ICUadmittance*	20 (54)	0 (0)	6 (43)	3 (37)	0 (0)	5 (31)	9 (75)	46 (44)
CVC*	29 (78)	1 (7)	10 (71)	5 (62)	1 (25)	11 (69)	11 (92)	68 (64)
TPN*	31 (81,6)	1 (7)	10 (71)	4 (50)	0 (0)	11 (69)	10 (83)	65 (62)
RRT*	6 (16)	0 (0)	2 (14)	0 (0)	0 (0)	2 (12)	6 (50)	16 (15)
Steroids*	10(27)	2 (14)	6 (43)	4 (50)	0 (0)	16 (37)	7 (58)	33 (32)
Immune supp.*	11 (30)	2 (14)	3 (21)	8 (100)	0 (0)	9 (56)	4 (33)	37 (35)
Leucopenia*	0 (0)	0 (0)	0 (0)	6 (75)	0 (0)	2 (12)	0 (0)	8 (7,6)
Diabetes*	3 (8,1)	3 (21,4)	3 (21,4)	2 (25)	1 (25)	4 (25)	1 (8,3)	17 (16)

*N (%),

# median (range).

Antiboitic days: total number of days with antibiotic treatment until days of candidemia.

IDU: Intravenous drug use. CVC: central venous catheter. TPN: Total parenteral nutrition. RRT: Renal replacement therapy.

## Discussion

The candidemia incidence of 2,6/100 000/year remain low compared with the Norwegian national incidence rate of 2,4/100 000/year for the preceding 13 years (1991–2003) [14]. Numbers are not completely comparable, as bone marrow and solid organ transplants are not accounted for by the present study, and these high risk patients could contribute to higher overall incidence on a national level. No major shift in the distribution of isolates was seen, in particular the dominance, even increase of the proportion of *C.albicans* (77%), was confirmed. Resistance patterns continue to be favourable with all *C.albicans* susceptible to fluconazole (table 1).

Absolute number of candidemias was highest in the medical department, but the incidence were higher in the departments of Gastro-surgery and Urology (table 2). Department of Cardiothoracic surgery counted 4/105 patients, all other specialities accounted for only 1 or 2 (Neurology) patients each. Patients admitted to medical wards present with more unclear and diverse conditions. We therefore attempted to group all candidemia patients independently from ward of admittance into clinical recognisable categories according to organ responsible for hospitalization, or specialty involved in management. Such grouping was readily achieved in 75% of patients (table 4). More than half (58%) could be categorized into established specialities; Gastroenterological surgery, Urology, Haematology and Neonatal medicine. Medical patients were more difficult to classify. Well recognised risk groups such as intravenous drug abusers and haematological malignancies comprised 10% of total, another 13% were categorised within a Pneumonia category. Of these, 64% were diagnosed in the ICU representing a subset of pneumonia's complicating other conditions such as cardiac arrest, non-resolving sepsis, and organ failure.

The relative importance of urology patients representing 14/105 (13%) of candidemias in the cohort and with an incidence proportion of 0, 46 (0, 24–0, 88) per 1000 patients admitted to the urology department, was unexpected. Although urogenital candidiasis is recognised, the risk of candiduric patients developing candidemia has been considered very low [17], [18]. A distinctive feature in urology patients was obstructive uropathy with a nephrostomy or JJ stent in 11/14 patients. Urology patients were, apart from the group intravenous drug abusers, also the only group were no candidemias were diagnosed in the ICU. The cumulative survival in the IDU and urology categories were also higher compared to the other categories (Fig. 1). 20 of 37 patients (54%) of the abdominal surgery category were diagnosed in the ICU, leaving almost half of the group diagnosed on the regular ward, as opposed to 9/12 (75%) diagnosed in the ICU in the group other surgery. This left only 3 non-abdominal, non-urology surgery patients diagnosed with candidemia in general surgical wards over the whole 11 year period, stressing the role of major and complicated surgery needing ICU surveillance for the development of candidemia. Not only abdominal surgery per se, but also ensuing complications were important for developing candidemia, as 60% of abdominal surgery patients had at least two surgical procedures performed, and 26% between 3 and 8. The role of complications in this group was also reflected in days on antibiotics prior to candidemia. With a median of 22 antibiotic days it was the highest of all groups. These lengthy courses most likely reflect a lack of control of underlying disease, as prolonged antibiotic therapy is usually not required for abdominal surgical infections. Long antibiotic courses prior to candedima were also observed in medical patients, though with a median of 11–17 days, shorter than abdominal surgery patients but comparable to all other groups.

The lack of patients with profound and long lasting neutropenia after allogenic bone-marrow transplant and acute leukaemia induction therapies may explain that trough 2005–2012 only 8 out of 2193 patients discharged with neutropenia as a main or co-diagnosis were diagnosed with candidemia. Six of these suffered from hematologic malignancies. The findings emphasize the far lower risk of candiemia following chemotherapy for solid tumours or less aggressive lymphoma chemotherapy, representing the great majority of neutropenic patients in this cohort.

With only 10 ICU beds out of a total of 650 hospital beds, ICU patients accounted for 46% of all diagnosed candidemia's. Mean SAPS II score in the group was 49, which is higher than the mean SAPS II of 40 that has been the average in our ICU over many years. The increase in predicted mortality in this SAPS score interval is steep, thus even within ICU patients candidemia patients represent a subgroup with higher morbidity and worse prognosis. The numbers of ICU patients in this study was higher than in a Finnish and Scottish study [3], [13], [19] but in line with studies from other investigators, reporting 33%–55% of candidemia episodes found in the ICU [20], [21]. These differences may reflect variations in specialities represented in the different settings, especially solid organ and bone marrow transplantation activity.

As reported in other studies [22], we found high candidemia case fatality rates with, 30 days mortality of 49% and 1 year mortality of 64%. However, some observations raise questions concerning candidemia attributable mortality and causality. In particular, the observation that in 54% (28/52) of 30 days non-survivors candidemia was a terminal event, with cultures becoming positive on the day of death or thereafter, or the patient considered in the process of dying and all active treatment withdrawn. These findings would bias attributable mortality significantly. This also applies to the observation of advanced or incurable cancer in 54% (15/28) of terminal event candidemia patients as opposed to 9% (5/53) in 30 days survivors. Further, the median survival of advanced cancer patients in this latter group was only 82 days, emphasizing the truly dismal short-term prognosis of the combination of advanced cancer and candidemia. Arguably the argument could be made that earlier and more aggressive anti-fungal therapy could have prevented early deaths and prolonged survival. From our detailed reading of the medical records and involvement in treatment, we consider this a more unlikely explanation. Interestingly, 7 patients in the 30 days survivor group did not receive treatment due to unrecognized positive blood cultures. Three of these had major but successfully treated gastro surgical conditions (necrotizing cholecystitis, necrotizing pancreatitis, rectum perforation), two had minor urological conditions, (bladder stone, catheter occlusion), one drug abuser left hospital refraining treatment, and one patient was successfully operated with hemicraniectomy following cerebral infarction. Follow up medical history did not reveal any medical consequences of neglected treatment. A possible explanation for the benign outcome could be a transient nature of these candidemia's as opposed to a situation with continuous seeding form major extra vascular foci, or continuous seeding from intravascular devices. These cases do however underline the importance of resolving underlying medical problems for the outcome of candidemia. Thus in 35/105 (33%) of the whole cohort, lack of candidemia treatment seems not to have had an influence on mortality or morbidity.

## Conclusions

In summary, the epidemiology of candidemia in this 11 years survey in a secondary hospital setting seems largely unchanged compared to the national incidence in the previous 13 years period. No shift towards more non-albicans candidemias was observed. In a general secondary referral hospital, urology patients, and in particular patients with obstructive uropathy may be an under-recognised risk group, and the risk for neutropenic non-haematology patients may be overestimated. ICU patients and patients with major complicated abdominal surgery constitute the majority of patients. Candidemia seems to be a terminal event in a significant proportion of patients, and one year mortality suggests that an episode of candidemia is an ominous sign. However, without major underlying disease, candidemia may be a more benign condition than suggested by the crude candidemia associated mortality.
